# Investigation of Oral Health in Children from Urban Slums of Nairobi, Kenya

**DOI:** 10.3390/dj12070211

**Published:** 2024-07-10

**Authors:** Gianni Di Giorgio, Simona De Pasquale, Enrico Battaglia, Giulia Zumbo, Cristina Mollica, Rita D’Ecclesia, Antonella Polimeni, Maurizio Bossù

**Affiliations:** 1 Department of Oral and Maxillofacial Sciences, Pediatric Dentistry Postgraduate School, Sapienza University of Rome, Via Caserta, 6, 00185 Rome, Italy; gianni.digiorgio@uniroma1.it (G.D.G.); antonella.polimeni@uniroma1.it (A.P.); maurizio.bossu@uniroma1.it (M.B.); 2 Viving APS, Piazza G. Winckelmann 5, 00162 Rome, Italy; simondepa1991@gmail.com (S.D.P.); batenr@gmail.com (E.B.); 3 Department of Statistical Sciences, Sapienza University of Rome, 00185 Rome, Italy; cristina.mollica@uniroma1.it (C.M.); rita.decclesia@uniroma1.it (R.D.)

**Keywords:** pediatric dentistry, slums, Nairobi, Kenya, oral health, observational study, caries, MIH

## Abstract

For children living in the urban slums of Nairobi (Kenya), primary health conditions are not guaranteed, and oral diseases add further concern at social and institutional levels beyond the general poverty conditions. This study aims at determining the factors that influence the oral health status of children living in Nairobi slums. A cross-sectional study on school-aged subjects was conducted in June 2022 in three urban slum areas of Nairobi through a pediatric dental screening. The PI (Plaque Index), CPITN (Community Periodontal Index of Treatment Needs), and dmft (decayed, missing, and filled teeth index—deciduous) were considered as primary outcomes of dental health. Multivariate statistical analysis, based on ordinal and zero-inflated negative binomial regression models, was conducted to identify determinants of the oral outcomes in a wide set of potential predictors. A sample of 359 children aged 2–17 was examined. The PI was significantly associated with age, the type of bite, and the use of a toothbrush. The CPITN is influenced by different types of malocclusions, abnormal frenulum, dental trauma, and fluorosis. Dietary habits were found to significantly impact the susceptibility to dental caries. Fluorosis and a dental visit in the last year were highlighted as risk and protective factors, respectively, against higher rates of caries. The oral health features of children living in Nairobi slums are differently affected by socio-demographic conditions, dietary habits, dental characteristics, and oral care practices.

## 1. Introduction

Kenya is a country where there is no National Health System and health care is usually fee-for-service. According to the forecasts of the United Nations and Population Pyramids (populationpyramids.net), the population in Africa is expected to increase from the current number of 1.1 billion to 4.3 billion by 2100. One picture emerges above all: 70% of Africans who move from the countryside to the city will not find space in more structured urban areas, resulting in people moving into slums. Within this vortex of growth and poverty, most people will not be able to afford their own medical costs [1]. 

For many rural communities in Kenya, dental care is non-existent due to difficulties with transportation and costs. In 2019, the reported number of registered dentists was 1288, resulting in a dentist:population ratio of 3:100,000 [2], with most dentists practicing in urban areas inhabited by only 20% of the population.

The use of fluoride has proven to be the most effective preventive public health measure against caries. However, here the cost of fluoride toothpaste is incredibly high, hindering widespread use. According to Goldman et al. [3], the poorest 30% of the UK population takes only 0.037 days of household expenditure to purchase the average annual dose of the cheapest toothpaste, compared to 10.75 days in Kenya.

Nairobi is the capital of Kenya with 5 million inhabitants, 60% of whom live in the 110 slums that surround the city center. The two slums of Nairobi, Korogocho and Dandora, arose during the 1960s and 1970s, respectively. They are inhabited by about 1.5 million people who live in dilapidated houses, and sheet metal shacks often without electricity, sewers, and running water.

The first (and only) Kenya National Oral Health Survey in 2015 reported a high oral disease burden. Notably, the prevalence of dental caries in children is 40%, gum disease is over 90%, and dental fluorosis is 41% [4]. This population is disadvantaged for several reasons, and many factors influence their quality of life, including food and nutrition, inadequate access to healthcare services, lack of proper oral hygiene habits, and non-intercepted harmful oral habits, but also predisposition related to the ethnic group.

The aim of this paper was to identify the factors explaining the oral health status in children living in urban slums of Nairobi, namely the plaque level, periodontal health status, and caries experience. A wide range of potential explanatory variables are taken into consideration, including socio-demographic conditions, dietary habits, dental occlusion characteristics, tooth damage, harmful oral habits, and practices of dental care. To the best of our knowledge, this is the first study on school-aged subjects in a developing country exploring multiple oral health features as primary outcomes and the effects of several predictors through an advanced multivariate analysis based on ordinal and zero-inflated negative binomial regression models [5].

## 2. Materials and Methods

In June 2022, a team of dentists from the Pediatric Dentistry Unit of Sapienza University of Rome conducted an oral screening at three private educational institutions in Nairobi (Kenya) in the context of the humanitarian activities of Alice For Children (https://aliceforchildren.it, accessed last on 4 July 2024) and Viving (https://www.viving.it, accessed last on 4 July 2024) associations, which operate in favor of local fragile young people.

A dental clinical evaluation was performed on the attendees of the schools in the slums of Korogocho and Dandora (St. Claire Primary School and Grapesyard Education Centre, respectively) and children hosted in the foster home Alice Village located in Utawala, a residential district of the city. Age below 18 and formal enrollment in the above institutions were the only sample selection criteria. These criteria were chosen in order to maximize the participation in the screening by younger subjects, compatible with the limited time frame of the mission and the available resources for humanitarian activities. Written informed consent for the visit was previously acquired through paper forms signed by the children’s local guardians.

The study implemented several measures: a thorough informed consent process with social workers to ensure participants and their guardians fully understood this study; strict confidentiality with anonymized data; risk minimization through comfortable examinations and prompt addressing of any urgent needs; involvement of experienced and calibrated clinicians; ongoing ethical oversight and monitoring; and arrangements for participant feedback and support during and after this study.

The entire study was conducted in accordance with the principles of the Declaration of Helsinki. Given the established relationships, the immediate need to conduct oral health examinations as part of ongoing humanitarian activities, and the limited timeframe of the mission, it was necessary to proceed with data collection promptly to identify dental needs. Recognizing the importance of ethical oversight, retrospective ethical approval was obtained from the Institutional Board of Sapienza University of Rome at the Faculty Council meeting held on 21 December 2022 (protocol n° 0000159/1601-23). This retrospective approval confirmed that this study adhered to all ethical requirements.

During the screening, an extensive and accurate oral visit was performed on each subject, and related data were collected by four independent, experienced, and calibrated clinicians according to the ICDAS (International Caries Detection and Assessment System) recommendations [6]. The clinical examination was carried out in a room with natural daylight with the children lying on tables. Visibility was enhanced with the use of head torches. The tools used for the screening were disposable dental kits composed of mirrors, probes, and tongue depressors. Cotton rolls were used to control moisture during the examination.

**Figure 1:**
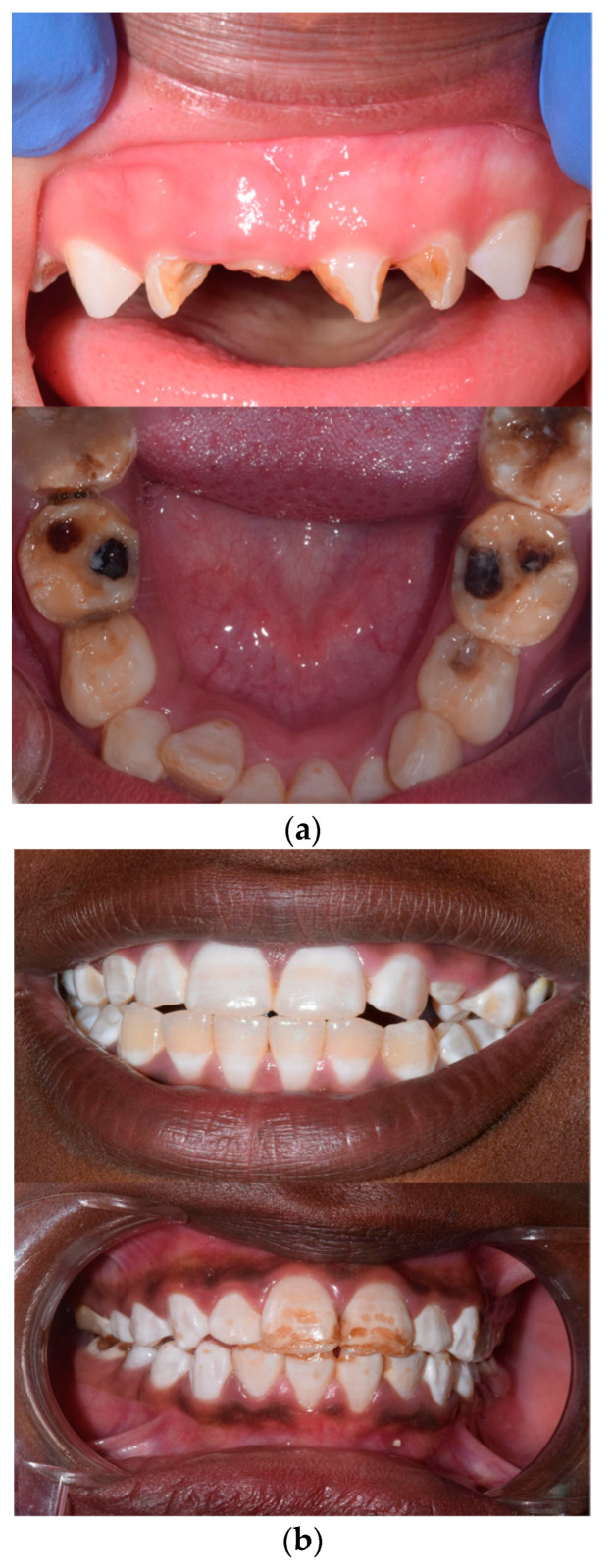
Some cases of caries (**a**) and fluorosis (**b**).

The assessment of periodontal status and diagnosis of caries were performed in accordance with the WHO’s Oral Health Survey Basic Methods [7]. Additionally, clinicians used their own clinical judgment to identify and differentiate the presence of caries, fluorosis [8], and MIH (Molar Incisor Hypomineralization) as shown in Figure 1. Additionally, anamnestic data were collected and double-checked with the help of local volunteers, school nurses, and teachers, who also mediated for non-English-speaking children.

A regression modeling analysis was designed to determine the factors affecting various aspects of the oral health condition by specifically considering three fundamental outcomes as the responses of interest:

1. The Loe–Silness Plaque Index (PI) is represented as an ordinal response with four levels: 1 = no plaque in the gingival area; 2 = a film of plaque adhering to the free gingival margin and adjacent area of the tooth, recognized by running a probe across the tooth surface (mild inflammation); 3 = moderate accumulation of soft deposits, which can be seen by the naked eye (moderate inflammation); and 4 = abundance of soft matter within the gingival pocket and/or on the gingival margin and adjacent tooth surface (severe inflammation) [9].
2. The Community Periodontal Index of Treatment Needs (CPITN) is represented as an ordinal response with three levels: 1 = normal gingiva, 2 = bleeding, and 3 = calculus. Since the probe cannot be used on the pediatric population, in the present study, we considered the simplified version of the original 5-level CPITN proposed in [10].
3. The decayed, missing, and filled teeth index (dmft) in primary dentition is represented as a numerical count response. The dmft index is a valuable measure for determining and monitoring oral health status in a community. It is computed as the total number of decayed, missing, and filled deciduous teeth due to caries [11]. The DMFT index for the permanent teeth was not taken into consideration since in most of the young participants the first molars were not or were only partially erupted, limiting the possibility of assessing the role of the explanatory variables on this type of dentition.

A large number of variables were considered as potential predictors of the three oral outcomes in the regression models:

- *Sociodemographic characteristics*: gender, age, and the educational institution attended.
- *Dietary habits*: consumption of sweets, juices, and fruit.
- *Practices of dental care*: use of a toothbrush and visit by a dentist in the last 12 months.
- *Oral characteristics and habits*: molar class, type of bite, overjet, crossbite, presence of abnormal frenulum, signs of dental trauma, fluorosis, MIH, and presence of bad oral habits.

Fluorosis is defined as the presence of developmental disturbance of dental enamel caused by successive exposures to high concentrations of fluoride during tooth development, leading to enamel with lower mineral content and increased porosity [12], whereas MIH was defined as the presence of developmental defects of enamel in molars and incisors, with or without association of post-eruptive breakdown [13].

First, a bivariate association analysis between each covariate and the three outcomes of interest was preliminarily carried out by means of the Kruskal–Wallis and Kendall rank correlation tests. Then, as a confirmatory approach to the exploratory bivariate associations, we performed a multivariate regression analysis separately for each oral outcome. Specifically, we estimated ordinal logistic regression models for predicting PI and CPITN levels, and the zero-inflated negative binomial regression model (ZINB) for the dmft values. The use of the ZINB is motivated by the possibility of jointly accounting for overdispersion and excess zeros, which characterize the observed dmft counts. For further evidence on the effectiveness of the ZINB in modeling dmft indices compared to other count regression models, see [14,15]. For the ZINB, the same covariates were used for the count and the zero-inflation model components. Finally, the optimal sets of covariates for the three regression models were identified with the backward selection procedure [16] based on the minimization of the AIC (Akaike’s Information Criterion) [17].

All the tests performed in the statistical analysis were two-sided and a *p*-value lower than 0.05 was considered as evidence of a statistically significant association. The statistical procedures were performed with the R software (version R Core Team (2023)). Specifically, the ordinal and ZINB regression models were estimated with the *polr* and *zeroinfl* functions from the MASS and pscl packages, respectively.

## 3. Results

A total of 374 children were initially screened by the team of dentists. However, due to difficulties in recovering reliable information, for example, on the exact age of orphan children or oral practices (such as bad habits, past dental visits, or use of a toothbrush), some missing values occurred in the data. Since the estimation of regression models requires the observation of all outcome variables and predictors, 15 children with an incomplete individual profile were necessarily excluded from the analysis. Hence, the final sample of the cross-sectional study was composed of 359 children, covering 17% of all 2072 children attending the educational institutions subject to the screening.

Descriptive statistics are presented in Table 1 as frequencies (%) for categorical variables and mean ± sd and range for numerical ones. The study participants were aged between 2 and 17 (7.2 ± 2.6 years old on average) and distributed in the three educational institutions of Nairobi as follows: 80% in the schools of Korogocho and Dandora (40% each), attended by children aged from 6 to 8 years old, and 20% in Alice Village, hosting a more heterogeneous age group. Concerning eating habits, the majority of children claimed not to consume sweets (58%) or juices (53%), whereas fruit consumption was widespread (78%). The orthodontic evaluation highlighted that most of the participants had a normal occlusion (83%). However, the most observed types of malocclusions were class III (13% vs. 4% of class II), open bite (29% vs. 3% deep), and augmented overjet (14% vs. 7% reduced). Nearly all of the sample had a normal frenulum (96%) and did not have any signs of fluorosis (93%), dental trauma (94%), or MIH (91%). Harmful oral habits (such as thumb sucking, tongue thrust swallowing, nail biting, etc.) were apparent in 24% of the screened subjects. Concerning oral hygiene and care, most of the participants reported using a toothbrush (79%), but a wide majority (82%) were not visited by a dentist in the last year.

**Figure 2:**
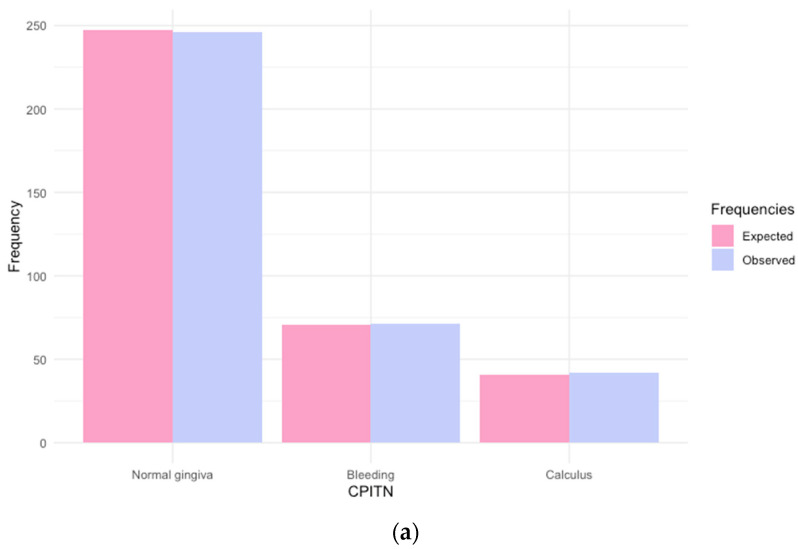

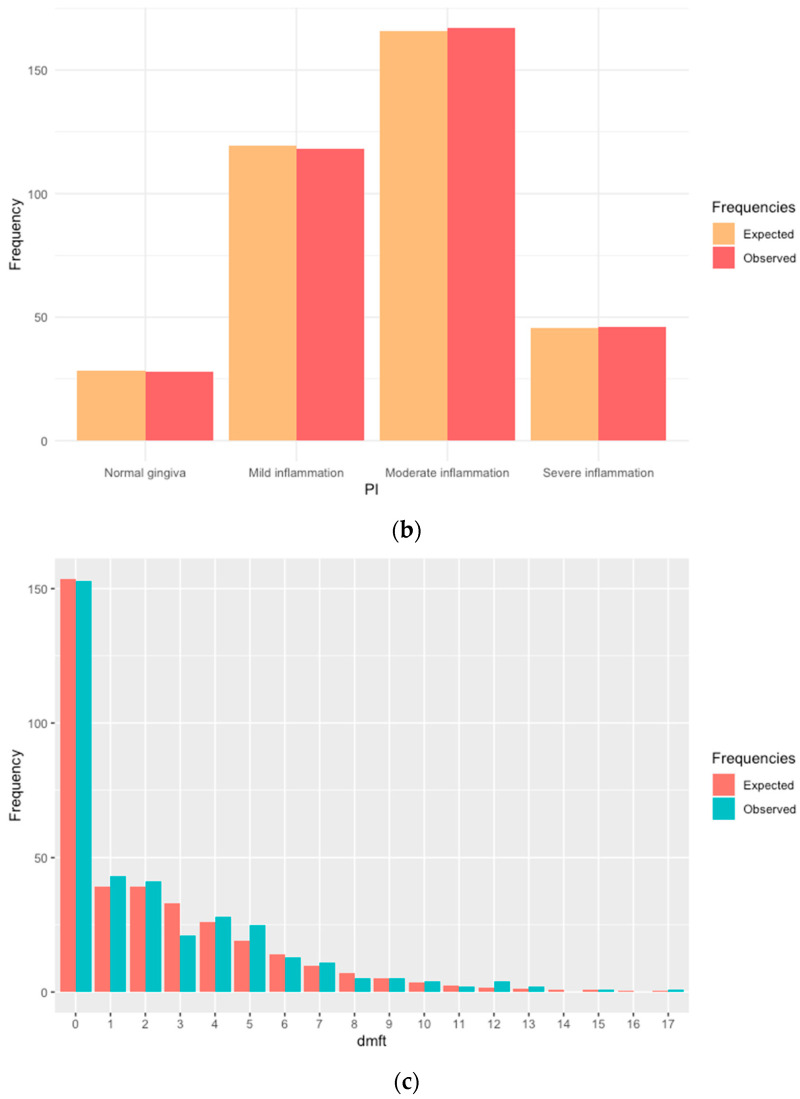
(**a**–**c**) Comparison between the observed frequencies of the dental outcomes and the estimated frequencies with regression models.

The frequency distributions of the three oral health outcomes are displayed in Figure 2. Dental plaque was observed in almost all the participants (92%), with moderate inflammation being predominant (46%). Gingivitis was detected in 32% of the sample. The prevalence of dental caries was 57%, with a mean dmft of 2.3 ± 3.1. Figure 2c shows that the distribution of the number of caries is characterized by a high degree of variability (overdispersion), with a major peak representing the 43% of participants with dmft = 0 and the remaining percentage of subjects with a number of caries spread over a wide range [0, 17].

The *p*-values of the bivariate association tests are reported in Table 2. From this preliminary analysis, we first remarked that statistically significant differences among the screening locations were found for all the outcomes considered. PI levels were also associated with the presence of MIH and older age; a significant relationship was highlighted between CPITN values and the presence of fluorosis as well as the use of the toothbrush, whereas the dmft was associated with the consumption of juices, fluorosis, dentist visit in the last year, and older age.

However, due to the lack of control for confounding effects, we stress that these results do not provide conclusive evidence for explaining oral health outcomes, but more reliable findings were obtained with regression analysis.

Regarding the ordinal regression model for the PI (Table 3), older subjects are at greater risk of gingivitis. There is also an indication that an abnormal frenulum could be associated with an increased PI since the corresponding *p*-value is close to the margin of significance. On the other hand, a deep bite is associated with a significantly lower level of gingivitis and also the use of a toothbrush emerges as a protective factor for the gingiva inflammation. Finally, the model indicates that children from the Korogocho location have significantly higher levels of plaque compared to the other screening locations.

The ordinal regression model for the CPITN pointed out several risk factors for periodontal disease (Table 4). In particular, the presence of fluorosis, abnormal frenulum, and dental trauma is associated with a significantly increased degree of gingivitis, as well as subjects with an open bite and a smaller overjet. The significance of age is at the boundary of the 0.05 critical threshold but, in line with the regression models for PI, age is suggested to be an additional risk factor. Differences among screening locations also emerge for the CPITN, with participants of the Alice Village characterized by significantly lower levels of the index, whereas subjects in the Dandora location are those with a significantly worse periodontal condition.

The estimates of the optimal ZINB for the dmft are displayed in Table 5, where the upper panel contains the results of the logistic regression for predicting excess zeros and the lower panel contains the results of the negative binomial regression for describing the count values.

The estimates of the zero-inflation part suggest that subjects who were visited by a professional dentist during the last year are significantly more likely to be caries-free. Analogous evidence emerges for those living in Alice Village. Finally, marginal evidence (*p*-value = 0.046) of a lower probability of being susceptible to caries is obtained for children with fluorosis. From the count regression part, we pointed out that the consumption of fruit and juices significantly increases the rate of caries. Moreover, children from the Dandora school exhibit significantly higher dmft values. However, differently from the estimate obtained with the zero-inflation part, the fluorosis condition is highlighted as a significant risk factor associated with the worst caries experience.

We finally assessed the goodness-of-fit by comparing the observed and expected frequencies under the estimated models. As apparent in Figure 2, the predicted frequencies are close to the observed ones for all the dental outcomes. We also remark on the ability of the ZINB to almost perfectly recover the observed zero counts (153), with an expected frequency equal to 154 against the remarkable underestimation (139) that would have been obtained with the ordinary negative binomial model. The significant result obtained for the Vuong test statistic (*p*-value < 0.001) further endorsed the better adequacy of the ZINB compared to the ordinary negative binomial regression.

## 4. Discussion

A wide range of studies have identified risk and protective factors for oral health by simply relying on a purely exploratory bivariate analysis [18,19]. Additionally, these works [20,21] overlook the control of confounding variables, which may lead to misleading results. In this study, we estimated the adjusted effect of a series of potential factors by implementing a multiple regression analysis for three main outcomes of the oral health condition, namely ordinal regression models for the PI and CPITN levels and the ZINB for the DMFT counts. The use of the ZINB has been promoted in the very recent literature on dental caries. To the best of our knowledge, this analysis represents one of the first applications of the ZINB to the caries experience of school-aged children from a developing country. All the estimated regression models were checked to provide an adequate fit and, interestingly, they highlighted how the three relevant features of dental health are differently impacted by socio-demographic characteristics, dietary habits, types of teeth malocclusion, harmful oral habits, dental damage, and oral care. Noteworthy, the results of our regression analysis show the limits of a conventional bivariate association approach to handle multiple explanatory variables, as highlighted by the differences between the significant findings returned by the two statistical analyses (Table 2 vs. Table 3, Table 4 and Table 5).

In the zero-inflation part of the model, fluorosis resulted as a protective factor against the development of caries, whereas in the count part, it is highlighted as a risk factor. This contrasting evidence could suggest that the relationship with the caries experience depends on the degree of severity of the fluorosis condition. Specifically, questionable and mild levels of fluorosis could have a preventive role and increase the probability of being caries-free, whereas higher levels could inflate the rate of caries. Nevertheless, we stress the ZINB provided an effective tool to establish the different effects of fluorosis on the susceptibility and intensity of the caries process that require further investigation.

These findings could be explained by the findings of Robinson et al. [22], who reported the effects of different fluoride intake on amelogenesis, manifesting at various levels from simple opaque white areas of enamel to enamel pitting. The latter may create a suitable niche for bacterial accumulation and, therefore, it is more likely for a dentition with severe fluorosis to be affected by caries [23].

From our study results, it emerges that children visited by a dentist in the year were the ones more likely to have caries. This is in contrast with other studies reporting significantly higher caries prevalence in children examined by a dentist at least once [24]. There is evidence that, in such conditions, children tend to visit the dentist when there is already a problem. Our findings can be justified by the fact that some children are already included in a prevention program with local dentists working at “Virginia’s Pediatric Dentist Unit” of the Ruaraka Uhai Neema Hospital in Nairobi, Kenya. A study conducted through the use of questionnaires in a school in the slum of Thane (India), on children aged 8–12, shows as well that oral health knowledge was poor at baseline; after a program of Oral Health Education, there was a significant improvement in the oral health knowledge and in the awareness about oral health [25].

The average value of the dmft that we observed in our study is in line with the statistics in [26], which reported an increase in the dmft index in children aged 3–5 old in Kenya from 1.5 in the 1980s to 2.95 in the 2000s, in accordance with another study conducted in the 1990s [27] where the mean dmft in a sample of 6- to 8-year-old children was 1.7.

We pointed out a difference between the three screening locations regarding the prevalence of caries and CPTIN: participants from Dandora were more affected by caries and had worse periodontal conditions, and those from Alice Village were more frequently caries-free and had significantly lower levels of periodontal indices.

We investigated the dietary habits of children in different locations and discovered that they are offered one meal a day at school in Dandora and Korogocho. At Alice Village, instead, they have a full diet from breakfast to dinner defined by a nutritionist from the Ruaraka Uhai Neema Hospital. Moreover, at Alice Village, children have the opportunity to brush their teeth after lunch while in other places they do not. A study conducted on 684 children from Nigerian slums observed that though the children infrequently consumed cariogenic meals, over a third of the children did not use fluoridated toothpaste while 96.5% had no Dental Home or previous dental visits. Dental caries, which was mainly untreated, was moderately prevalent among the children surveyed in this urban slum in Nigeria. There was also a significant association between the presence of caries in this population and low BMI [28].

A cross-sectional survey conducted in twelve slum clasts of Tongi, Bangladesh, confirms the previously reported data on socially deprived areas in other parts of the world, such as urban slums of Kenya and Nigeria. Also, in the slums of Tongi, a high level of dental diseases was identified, with poor periodontal and oral hygiene status and a high level of DMFT (mean DMFT 1.1 to 4.7). It was evident that populations living in the slum area were deprived of any basic interventional or preventive programs related to oral diseases [29].

Our research also indicates several practical directions to enhance dental outcomes in low-income countries for the young-age population. The results presented in this work, in fact, can guide in planning future actions involving oral hygiene awareness programs; organization of screening programs in collaboration with schools; strengthening of partnerships among schools, voluntary associations, and hospitals for the early detection of pathologies; and international collaboration among oral health institutions for the training of local doctors.

Another cross-sectional survey of 385 adults in the urban slums area of Rawalpindi, Islamabad region, Pakistan, revealed a high prevalence of oral disease among residents of urban slums, with a mean DMFT index of 8.91 + 7.63 and a mean CPITN index of 1.93 + 0.97 [30].

Periodontal disease was detected through bleeding on probing or the presence of calculus in 20% and 12% of the subjects, respectively. The first Kenyan National Oral Health Survey in 2015 reported a prevalence of 75.7% of gingival bleeding in children [4]. Another study [27] reported visible plaque in 75% of the participants. Finally, children from Korogocho are those with significantly higher levels of plaque.

When asked about ownership of a toothbrush, 21% of the screened participants were found not to have it. Some of them referred to using the toothbrush of a parent or a familiar, and others to using the chewing stick (mswaki). The prevalence of gingivitis is very high in our sample, with only 8% of children without plaque. This is in accordance with other studies [31]. Some investigations in Nairobi have examined the efficiency of mswaki and commercial toothbrushes on plaque removal, showing the mswaki as being less efficacious [32,33]. In a context where parents of children enter the landfill every day to earn the minimum necessary to keep their families alive (less than USD 2 a day) [1], buying toothpaste and a toothbrush seems something unthinkable. At the end of the screenings, a toothbrush and toothpaste were given to each child, along with oral hygiene instructions. During upcoming screenings to be conducted in the same schools, the effect of regular use of a toothbrush on the Plaque Index, dental caries, and periodontal index will be evaluated.

Deep bite emerges as a protective factor for gingiva inflammation, while open bite does not give statistically significant results. However, there are studies showing that occlusion characteristics and breathing type can affect oral status. In particular, mouth breathing and decreased upper lip coverage are associated with increased levels of plaque and gingival inflammation [34].

## 5. Conclusions

The oral health of children living in Nairobi slums is influenced by various socio-demographic factors, dietary habits, dental characteristics, and oral care practices. However, further exploration is needed to increase the overall understanding of the role of impactful variables on dental health conditions, particularly regarding the impact of fluoride concentrations.

Future studies on the determinants of dental health in children from developing countries could be further improved by enlarging the set of possible influencing factors. Indeed, some additional characteristics were collected during the screening activity, but these variables were ultimately omitted from the analysis due to different limitations, such as low sample variability or difficulties in recovering reliable information, especially from orphan or very young participants. Both issues may be addressed by increasing the number of participants, which could also allow us to better explore the role of those covariates that, in the present study, have been considered only in dichotomous form. In fact, differences in oral health outcomes among patients with distinct types of enamel defects, trauma, and damaging oral habits as well as among those reporting different frequencies of food consumption and dental care practices are expected, as already testified in other studies [20,21]. A further limitation of our study concerns the considered sample selection procedure. In fact, caution in extending the estimation results to the whole school-aged population in Nairobi must be considered since the sample was not obtained with a random sampling procedure. Additionally, future investigations should also involve public educational institutions to improve the representativeness of the young population in Nairobi.

Our research also indicates several practical directions to enhance dental outcomes in low-income countries for the young-age population. The results presented in this work, in fact, can guide in planning future actions involving (i) oral hygiene awareness programs; (ii) organization of screening programs in collaboration with schools, representing important reference institutions for young people, supported by the activity of voluntary associations to fight school drop-outs; (iii) strengthening of partnerships among schools, voluntary associations, and hospitals for the early detection of pathologies; and (iv) international collaboration among oral health institutions to favor scientific updating and on-site training of local doctors.

Our research was made possible thanks to the pediatric screenings and humanitarian activities coordinated by the international collaboration of Italian health and academic institutions with local organizations. An international effort to reduce the burden of dental diseases is testified also by the WHO, which first published its 2016 reference manual to help prevent and manage oral diseases at the primary healthcare level [35] and later its 2016–2025 strategy, setting some of the abovementioned actions as future objectives [36]. Moreover, some steps are being taken regarding oral health; in particular, the Kenyan government has taken action with the establishment of the Kenya National Oral Health Policy 2022–2030, which led to setting as a goal, among all, the decrease in the prevalence of dental caries among 5-year-old children from 46.3% in 2015 to 34.7% in 2030, and the prevalence of fluorosis in children from 41.7% in 2015 to 20.7% in 2030. These objectives will be pursued by implementing standardized school oral health programs as part of the Health Promoting Schools Initiative [4], supported by promising programs, objectives, and results already recently reported in the literature and also conducted in other countries [37,38,39].

## Figures and Tables

**Table 1:** Descriptive statistics for the socio-demographic and oral health characteristics of the *n* = 359 children included in this study.

Categorical Variables	*n* (%)
Gender: Male	158 (44)
Gender: Female	201 (56)
Educ. institution: Korogocho	144 (40)
Educ. institution: Dandora	143 (40)
Educ. Institution: Alice Home	72 (20)
Consumption of sweets: No	209 (58)
Consumption of sweets: Yes	150 (42)
Consumption of juices: No	189 (53)
Consumption of juices: Yes	170 (47)
Consumption of fruit: No	79 (22)
Consumption of fruit: Yes	280 (78)
Molar class: Class I	296 (83)
Molar class: Class II	15 (4)
Molar class: Class III	48 (13)
Type of bite: Normal	245 (68)
Type of bite: Open	104 (29)
Type of bite: Deep	10 (3)
Overjet: Normal	284 (79)
Overjet: Greater	49 (14)
Overjet: Smaller	26 (7)
Presence of crossbite: No	342 (95)
Presence of crossbite: Yes	17 (5)
Presence of abnormal frenulum: No	344 (96)
Presence of abnormal frenulum: Yes	15 (4)
Presence of fluorosis: No	332 (93)
Presence of fluorosis: Yes	27 (7)
Presence of MIH: No	326 (91)
Presence of MIH: Yes	33 (9)
Signs of dental trauma: No	344 (96)
Signs of dental trauma: Yes	15 (4)
Harmful oral habits: No	272 (76)
Harmful oral habits: Yes	87 (24)
Use of toothbrush: No	76 (21)
Use of the toothbrush: Yes	283 (79)
Dentist visit in the last year: No	295 (82)
Dentist visit in the last year: Yes	64 (18)
PI: Absence of plaque	28 (8)
PI: Mild inflammation	118 (33)
PI: Moderate inflammation	167 (46)
PI: Severe inflammation	46 (13)
CPITN: Normal gingiva	246 (68)
CPITN: Bleeding	71 (20)
CPITN: Calculus	42 (12)
**Numerical Variables**	**mean ± sd [Range]**
Age (years)	7.8 ± 2.6 [2, 17]
dmft	2.3 ± 3.1 [0, 17]

Descriptive summaries are expressed as frequencies and percentages (*n* and %) and mean ± sd and range for categorical and numerical variables, respectively.

**Table 2:** The *p*-values of the association tests between each predictor and outcome variable.

Variable	PI	CPITN	dmft
Gender	0.349	0.705	0.117
Educ. institution	**<0.001**	**<0.001**	**<0.001**
Consumption of sweets	0.501	0.876	0.107
Consumption of juices	0.787	0.930	**<0.001**
Consumption of fruit	0.485	0.577	0.069 *
Molar class	0.596	0.234	0.344
Type of bite	0.114	0.121	**0.044**
Overjet	0.331	0.185	0.426
Presence of crossbite	0.820	0.252	0.847
Presence of abnormal frenulum	0.100	0.076 *	0.148
Presence of fluorosis	0.826	**0.035**	**0.037**
Presence of MIH	**0.039**	0.884	0.516
Signs of dental trauma	1.000	0.051 *	0.841
Harmful oral habits	0.166	0.648	0.851
Use of the toothbrush	0.396	**0.019**	0.055 *
Dentist visit in the last year	0.927	0.449	**0.005**
PI	-	**<0.001**	0.359
CPITN	**<0.001**	-	**<0.001**
dmft	0.359	**<0.001**	**-**
Age (years)	**0.036**	0.690	**<0.001**

Significant *p*-values at level 0.05 are highlighted in bold. Those significant at level 0.10 are indicated with the * symbol.

**Table 3:** Results of the optimal ordinal logistic regression model obtained with the backward variable selection procedure to predict the PI.

Variable	OR	95%CI	*p*-Value
Age (years)	**1.293**	**[1.170, 1.431]**	**<0.001**
Educ. institution: Dandora	**0.315**	**[0.186, 0.527]**	**<0.001**
Educ. institution: Alice Home	**0.222**	**[0.109, 0.448]**	**<0.001**
Type of bite: Open	0.947	[0.600, 1.496]	0.817
Type of bite: Deep	**0.166**	**[0.050, 0.536]**	**0.003**
Presence of abnormal frenulum: Yes	2.714	[0.974, 7.749]	0.058 *
Presence of MIH: Yes	1.694	[0.848, 3.408]	0.136
Use of the toothbrush: Yes	**0.467**	**[0.263, 0.825]**	**0.009**
CPITN: Bleeding	**2.197**	**[1.273, 3.816]**	**0.005**
dmft	**1.150**	**[1.067, 1.240]**	**<0.001**

OR = odds ratio. CI = confidence interval. *p*-value refers to the two-sided tests of significance of the estimated adjusted odds ratios in the best ordinal logistic regression model with PI as the outcome variable. Significant *p*-values at level 0.05 are highlighted in bold. Those significant at level 0.10 are indicated with the * symbol.

**Table 4:** Results of the optimal ordinal logistic regression model obtained with the backward variable selection procedure to predict the CPITN.

Variable	OR	95%CI	*p*-Value
Age (years)	1.138	[0.998, 1.301]	0.055 *
Educ. institution: Dandora	**2.073**	**[1.214, 3.579]**	**0.008**
Educ. institution: Alice Home	**0.316**	**[0.107, 0.856]**	**0.029**
Type of bite: Open	**1.862**	**[1.042, 3.325]**	**0.035**
Type of bite: Deep	1.818	[0.416, 7.080]	0.399
Overjet: Greater	1.422	[0.702, 2.804]	0.317
Overjet: Smaller	**2.477**	**[0.990, 6.034]**	**0.048**
Presence of abnormal frenulum: Yes	**2.989**	**[1.016, 8.563]**	**0.042**
Presence of fluorosis: Yes	**3.062**	**[1.272, 7.351]**	**0.012**
Signs of dental trauma: Yes	**3.822**	**[1.162, 11.928]**	**0.022**
Harmful oral habits: Yes	0.596	[0.307, 1.128]	0.118
PI: Mild inflammation	6.182	[1.118, 12.697]	0.090 *
PI: Moderate inflammation	**13.933**	**[2.590, 26.627]**	**0.013**
PI: Severe inflammation	**18.181**	**[3.106, 35.684]**	**0.008**
dmft	**1.124**	**[1.039, 1.217]**	**0.004**

OR = odds ratio. CI = confidence interval. *p*-value refers to the two-sided tests of significance of the estimated adjusted odds ratios in the best ordinal logistic regression model with CPITN as the outcome variable. Significant *p*-values at level 0.05 are highlighted in bold. Those significant at level 0.10 are indicated with the * symbol.

**Table 5:** The results of the optimal zero-inflation negative binomial regression model for the deciduous dmft obtained with the backward variable selection procedure.

**Zero-Inflation Part (Logistic Regression Model)**			
**Variable**	**OR**	**95%CI**	***p*** **-Value**
Age (years)	1.163	[0.998, 1.354]	0.052 *
Educ. institution: Dandora	0.927	[0.469, 1.834]	0.828
Educ. institution: Alice Home	**3.033**	**[1.120, 8.212]**	**0.029**
Presence of fluorosis: Yes	**2.882**	**[1.016, 8.177]**	**0.047**
Dentist visit in the last year: Yes	**3.143**	**[1.477, 6.687]**	**0.003**
**Count Part (Negative Binomial Regression Model)**			
**Variable**	**IRR**	**95%CI**	***p*** **-Value**
Age (years)	**0.866**	**[0.802, 0.935]**	**<0.001**
Educ. institution: Dandora	**1.363**	**[1.079, 1.722]**	**0.009**
Educ. institution: Alice Home	1.137	[0.726, 1.780]	0.574
Consumption of juices: Yes	**1.330**	**[1.042, 1.668]**	**0.014**
Consumption of fruit: Yes	**1.339**	**[1.003, 1.788]**	**0.048**
Presence of fluorosis: Yes	**1.799**	**[1.072, 3.018]**	**0.026**

OR = odds ratio. IRR = incidence rate ratio. CI = confidence interval. *p*-value refers to the two-sided tests of the significance of the regression coefficients. Significant *p*-values at level 0.05 are highlighted in bold. Significant *p*-values at level 0.10 are indicated with the * symbol.

## Data Availability

The data that support the findings of this study are not publicly available due to their containing information that could compromise the privacy of the research participants; however, these data are available upon request to the corresponding author [GZ].

## References

[1] Diego M. (2017). Exploding Africa.

[2] Ratio of Registered Dentists to 100,000 Population in Kenya 2015–2020. Published by Lars Kamer, 13 December 2021. https://www.statista.com/statistics/1240318/ratio-of-registered-dentists-to-100-000-population-in-kenya/.

[3] Goldman A.S., Yee R., Holmgren C.J., Benzian H. (2008). Global affordability of fluoride toothpaste. Glob. Health.

[4] Kenya National Oral Health Policy 2022–2030, Ministry of Health Republic of Kenya, April 2022. https://www.afro.who.int/countries/kenya/publication/kenya-national-oral-health-policy-2022-2030.

[5] Demarchi P., Garbarino F., Mascolo A., Silvestrini Biavati F., Ugolini A. (2023). Fluorosis and Oral Health Status in Adolescents Living in a High-Fluoride Groundwater Area: A Case Study of Nairobi Suburbs (Kenya). Appl. Sci..

[6] Pitts N.B., Ismail A.I., Martignon S., Ekstrand K., Douglas G.V., Longbottom C. (2014). ICCMS™ Guide for Practitioners and Educators.

[7] World Health Organization (2013). Oral Health Surveys: Basic Methods.

[8] Dean H.T. (1942). The investigation of physiological effects by the epidemiological method. Am. Assoc. Adv. Sci..

[9] Löe H. (1967). The Gingival Index, the Plaque Index and the Retention Index Systems. J. Periodontol..

[10] Guidelines for Periodontal Screening and Management of Children and Adolescents under 18 Years of Age. 2021. British Society of Periodontology and Implant Dentistry and British Society of Paediatric Dentistry. https://www.bsperio.org.uk/professionals/publications.

[11] Moradi G., Mohamadi Bolbanabad A., Moinafshar A., Adabi H., Sharafi M., Zareie B. (2019). Evaluation of Oral Health Status Based on the Decayed, Missing and Filled Teeth (DMFT) Index. Iran J. Public Health.

[12] Abanto Alvarez J., Rezende K.M., Marocho S.M., Alves F.B., Celiberti P., Ciamponi A.L. (2009). Dental fluorosis: Exposure, prevention and management. Med. Oral Patol. Oral Cir. Bucal..

[13] Krishnan R., Ramesh M. (2014). Molar incisor hypomineralisation: A review of its current concepts and management. SRM J. Res. Dent. Sci..

[14] Lewsey J., Thomson W. (2004). The utility of the zero-inflated Poisson and zero-inflated negative binomial models: A case study of cross-sectional and longitudinal DMF data examining the effect of socioeconomic status. Community Dent. Oral Epidemiol..

[15] Solinas G., Campus G., Maida C., Sotgiu G., Cagetti M.G., Lesaffre E., Castiglia P. (2009). What statistical method should be used to evaluate risk factors associated with dmfs index? Evidence from the National Pathfinder Survey of 4-year-old Italian children Community. Dent. Oral Epidemiol..

[16] Chowdhury M.Z.I., Turin T.C. (2020). Variable selection strategies and its importance in clinical prediction modelling. Fam. Med. Community Health.

[17] Akaike H. (1998). Information theory and an extension of the maximum likelihood principle. Selected Papers of Hirotugu Akaike.

[18] Ngatia E.M., Imungi J.K., Muita J.W., Nganga P.M. (2001). Dietary patterns and dental caries in nursery school children in Nairobi, Kenya. East Afr. Med. J..

[19] Wakhungu H.K., Were G.M., Serrem C.A., Kibosia C.J. (2020). Dietary Intake and Prevalence of Dental Caries among Five-Year-Old Children in Urban and Rural Areas of Uasin-Gishu County, Kenya. Eur. J. Agric. Food Sci..

[20] Johansson I., Holgerson P.L., Kressin N.R., Nunn M.E., Tanner A.C. (2010). Snacking habits and caries in young children. Caries Res..

[21] Okemwa K.A., Gatongi P.M., Rotich J.K. (2010). The oral health knowledge and oral hygiene practices among primary school children age 5-17 years in a rural area of Uasin Gishu district, Kenya. East Afr. J. Public Health.

[22] Robinson C., Connell S., Kirkham J., Brookes S.J., Shore R.C., Smith A.M. (2004). The Effect of Fluoride on the Developing Tooth. Caries Res.

[23] Arheiam A., Aloshiby A., Gaber A., Fakron S. (2022). Dental Fluorosis and Its Associated Factors Amongst Libyan Schoolchildren. Int. Dent. J..

[24] Zumpe L., Bensel T., Wienke A., Mtaya-Mlangwa M., Hey J. (2021). The Oral Health Situation of 12-Year-Old School Children in the Rural Region of Ilembula in Southwestern Tanzania: A Cross-Sectional Study. Int. J. Environ. Res. Public Health.

[25] Tirpude V., Gailot A., Singh R., Bajaj N., Fernandes G. (2018). The Impact of Oral Health Education on Children from the Slums of Thane: A Survey Study. Int. J. Sci. Res. Sci. Technol..

[26] Macigo F.G., James R.M., Ogunbodede E., Gathece L.W. (2016). Sugar consumption and dental caries experience in Kenya. Int. Dent. J..

[27] Ng’ang’a P.M., Valderhaug J. (1992). Dental caries in primary school children in Nairobi, Kenya. Acta Odontol. Scand..

[28] Olatosi O.O., Oyapero A., Ashaolu J.F., Abe A., Boyede G.O. (2022). Dental caries and oral health: An ignored health barrier to learning in Nigerian slums: A cross sectional survey. PAMJ-ONE Health.

[29] Hannan M.A., Chowdhury M.T., Khan M.A., Chowdhury A.F., Shahidullah K.M., Saha A.K., Anjum A. (2014). Prevalence of Gingivitis, Plaque accumulation and Decayed, Missing and Filled Teeth among slum population in Bangladesh. Bangladesh Med. Res. Counc. Bull..

[30] Habib M.F., Mahmood H., Khizar A., Idrees S., Pervaiz F., Khan J. (2022). Oral Health Status and Oral Hygiene Practices among Urban Slum Dwellers in Rawalpindi, Islamabad, Pakistan: Oral Health and Hygiene Practices among Urban Slum Dwellers. Pak. J. Health Sci..

[31] Owino R.O., Masiga M.A., Ng’ang’a P.M., Macigo F.G. (2010). Dental caries, gingivitis and the treatment needs among 12-year-olds. East Afr. Med. J..

[32] Ng’ang’a P.M., Valderhaug J. (1991). Oral hygiene practices and periodontal health in primary school children in Nairobi, Kenya. Acta Odontol. Scand..

[33] Ndung’u F.L., Kaimenyi J.T., Arneberg P., Muthami L.N. (1990). A comparative study of the efficacy of plaque control by a chewing stick and a tooth brush. East Afr. Med. J..

[34] Wagaiyu E.G., Ashley F.P. (1991). Mouthbreathing, lip seal and upper lip coverage and their relationship with gingival inflammation in 11-14 year-old schoolchildren. J. Clin. Periodontol..

[35] World Health Organization, Regional Office for Africa (2016). Promoting Oral Health in Africa: Prevention and Control of Oral Diseases and Noma as Part of Essential Noncommunicable Disease Interventions.

[36] Regional Committee for Africa, 66 (2016). Regional Oral Health Strategy 2016–2025: Addressing Oral Diseases as Part of Noncommunicable Diseases: Report of the Secretariat. World Health Organization, Regional Office for Africa. https://apps.who.int/iris/handle/10665/250994.

[37] Abuhaloob L., Petersen P.E. (2023). Health-Promoting Schools Project for Palestine Children’s Oral Health. Int. Dent. J..

[38] Chher T., Hak S., Courtel F., Durward C. (2009). Improving the provision of the Basic Package of Oral Care (BPOC) in Cambodia. Int. Dent. J..

[39] Turton B., Patel J., Sieng C., Tak R., Durward C. (2021). School Based Tooth Brushing and Annual Silver Diammine Fluoride Application as a Highest Priority Package for Achieving Universal Oral Health Care for Cambodian Children. Front. Oral. Health.

